# Gallic Acid Alleviates Neuropathic Pain Behaviors in Rats by Inhibiting P2X7 Receptor-Mediated NF-κB/STAT3 Signaling Pathway

**DOI:** 10.3389/fphar.2021.680139

**Published:** 2021-08-25

**Authors:** Runan Yang, Zijing Li, Yuting Zou, Jingjian Yang, Lin Li, Xiumei Xu, Günther Schmalzing, Hong Nie, Guilin Li, Shuangmei Liu, Shangdong Liang, Changshui Xu

**Affiliations:** ^1^ Neuropharmacology Laboratory of Physiology Department, Medical School of Nanchang University, Nanchang, China; ^2^ Jiangxi Provincial Key Laboratory of Autonomic Nervous Function and Disease, Nanchang, China; ^3^ Undergraduate Student at the Medical School of Nanchang University, Nanchang, China; ^4^ Institute of Clinical Pharmacology, RWTH Aachen University, Aachen, Germany; ^5^ Guangdong Province Key Laboratory of Pharmacodynamic Constituents of TCM and New Drugs Research, College of Pharmacy, Jinan University, Guangzhou, China

**Keywords:** neuropathic pain, chronic constriction injury, P2X7 receptor, gallic acid, dorsal root ganglia

## Abstract

Neuropathic pain is a complex disease with high incidence. Adenosine triphosphate (ATP) and its activated P2X7 receptor are involved in the signal transmission of neuropathic pain. Gallic acid (3,4,5-trihydroxybenzoic acid) is a traditional Chinese medicine obtained from natural plants that exhibit anti-inflammatory, analgesic, and antitumor effects. However, the underlying mechanism for gallic acid in analgesia remains unknown. This study aims to reveal how gallic acid alleviates neuropathic pain behaviors in a rat model with chronic constriction injury (CCI). Real-time PCR, western blotting, double-label immunofluorescence, molecular docking, and whole-cell patch clamp technology were used to explore the therapeutic action of gallic acid on neuropathic pain. The results showed that after CCI rats were treated with gallic acid for 1 week, the mechanical withdrawal threshold and thermal withdrawal latency were increased, accompanied by inhibition of the upregulated expression of P2X7 and TNF-α at both mRNA and protein levels, and reduced NF-κB and phosphorylated-STAT3 in the dorsal root ganglia. At the same time, gallic acid significantly decreased the coexpression of P2X7 and glial fibrillary acidic protein in the dorsal root ganglia. In addition, gallic acid could suppress ATP-activated current in human embryonic kidney 293 (HEK293) cells transfected with the plasmid expressing P2X7 but had no effect on ATP activation current of P2X7-mutant plasmid (with the point mutation sequence of the key site where gallic acid binds to the P2X7 receptor). Therefore, our work suggests that gallic acid may alleviate neuropathic pain in CCI rats by inhibiting the P2X7 receptor and subsequent activation of the TNF-α/STAT3 signaling pathway.

## Introduction

The latest definition for pain by the International Association for the Study of Pain (IASP) is displayed as an unpleasant feeling and emotional experience, which is related to actual or potential tissue injury (Raja et al., 2020). Neuropathic pain is attributed to pathological changes or injuries of the somatosensory nervous system, which is commonly and easily disabled (Calvo et al., 2019). Neuropathic pain can cause the activation of satellite glial cells in dorsal root ganglia (DRG) and promote signal transduction between neuronal synapses and the release of cytokines, chemokines, and various inflammatory factors, eventually leading to an increase in the abnormal discharge of neurons and resulting in hyperalgesia or allodynia (Liu et al., 2020). Neuropathic pain is a complex heterogeneous syndrome that makes treatment very difficult.

Tumor necrosis factor-α (TNF-α) is a pleiotropic inflammatory factor (Kalliolias and Ivashkiv, 2016). When the extracellular adenosine triphosphate (ATP) concentration increases, the P2X7 receptor is activated and acts on TNF-α converting enzyme (TACE). Sheared membrane-bound TNF-α becomes soluble free TNF-α, which may induce inflammation and neuropathic pain (Gogoi et al., 2020). Signal transducer and activator of transcription 3 (STAT3) can be activated by different cytokines. There is evidence that the TNF-α/STAT3 signaling pathway is activated in neuropathic pain (Ding et al., 2019). In a rat model of neuropathic pain established by chronic constriction injury (CCI), TNF-α can activate nuclear factor kappa-B (NF-κB), and activation of the NF-κB/STAT3 signaling pathway may participate in pain regulation (Guo et al., 2018; Chu et al., 2020).

Purinergic receptors include P1 and P2 subfamilies. P2 subfamily contains P2X (1–7) and P2Y_(1,2,4,6,11–14)_ receptors (Burnstock, 2017). When organs or tissues are damaged, ATP can be released into the inflammatory microenvironment. ATP acts as a signaling molecule to activate the P2X7 receptor through paracrine or autocrine signaling, which affects the homeostasis of the internal environment and the development of the inflammatory response (Di Virgilio et al., 2018). Thus targeting the P2X7 receptor may provide a new direction for anti-inflammatory therapy. Upregulation of glial fibrillary acidic protein (GFAP) expression indicates the activation of satellite glial cells (SGCs). When satellite glial cells are activated, large amounts of ATP and inflammatory cytokines can be released to activate the P2X7 receptor (Hu et al., 2020). Gallic acid (3,4,5-trihydroxybenzoic acid), as a kind of traditional Chinese medicine, can be obtained from gallnuts, sumac, and many other natural plants. Gallic acid has analgesic, anti-inflammatory, hypoglycemic, and lipid-lowering effects (Kong et al., 2018; Sohrabi et al., 2021). However, the underlying analgesic mechanism of gallic acid remains unknown. Therefore, the purpose of this study was to explore whether gallic acid could alleviate neuropathic pain behaviors in rats with CCI by inhibiting the P2X7 receptor mediated NF-κB/STAT3 signaling pathway.

## Materials and Methods

### Animal and CCI Model Establishment

**FIGURE 1:**
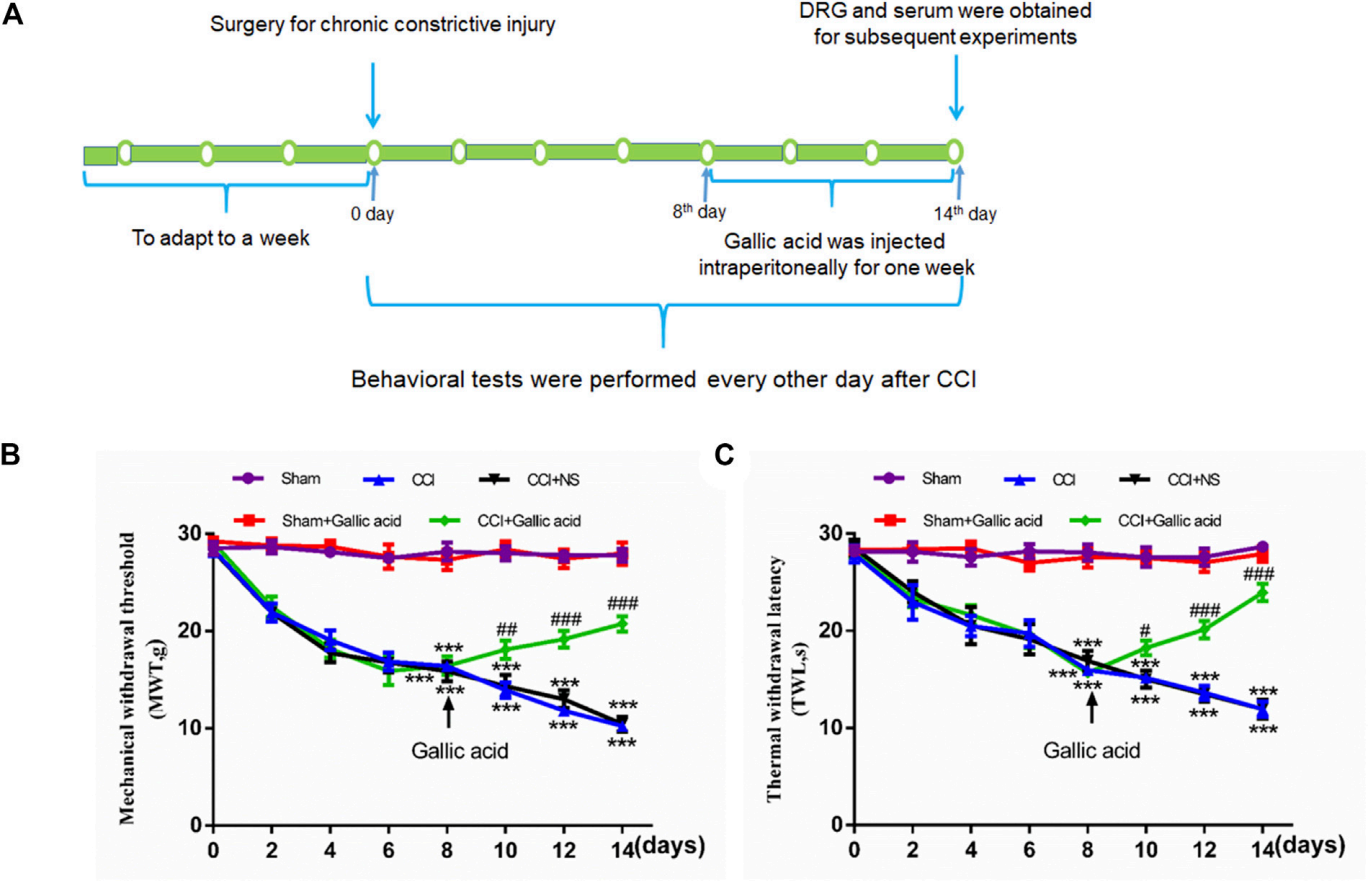
**Timeline of model establishment and pain behaviors results.(A).** Timeline of CCI modeling and drug treatment. **(B).** Changes in the mechanical withdrawal threshold (MWT) in rats subjected to gallic acid (time: F (7, 80) = 69.95, *p* < 0.001; Group: F (4, 80) = 353.8, *p* < 0.001; interaction: F (28, 80) = 12.78, *p* < 0.001). **(C).** Changes in the thermal withdrawal latency (TWL) in rats subjected to gallic acid (time: F (7, 80) = 9.974, *p* < 0.001; Group: F (4, 80) = 198.9, *p* < 0.001; interaction: F (28, 80) = 7.213, *p* < 0.001). Each group consisted of eight rats. Two-way ANOVA and Tukey’s honestly significant difference test were used to analyze the MWT and TWL. Data are presented as mean ± SEM. ***p < 0.001 versus the Sham group, ^#^ *p* < 0.05, ^##^ *p* < 0.01 and ^###^ *p* < 0.001 versus the CCI group.

Standard Sprague Dawley male rats, weighing 200–220 g, were provided by the medical animal center of Nanchang University. All experiments were conducted in accordance with the animal ethics committee of Nanchang University and followed the IASP guidelines on animal pain research. The CCI model was established according to a previously published method (Li et al., 2017). After anesthesia by pentobarbital sodium (40 mg/kg), the biceps femoris was bluntly dissected to expose the nerve, and the proximal end of the sciatic nerve was ligated 4 times with 4–0 intestinal thread. The distance between each node was 1 mm. The surgical treatment of the sham group was the same as that of the CCI group, except that the free nerves were not ligated. CCI model establishment and medication time are shown in Figure 1A.

To examine the effects of gallic acid on neuropathic pain, rats were randomly numbered and divided into five groups: sham operation group (Sham group), sham operation + gallic acid group (Sham + Gallic acid group), CCI model group (CCI group), CCI model + normal saline group (CCI + NS group), and CCI model + gallic acid group (CCI + Gallic acid group). Gallic acid (Shanghai Macklin Biological Co. Shanghai, China) was dissolved in normal saline. From the first day after CCI, the sham plus gallic acid group and the CCI plus gallic acid group were injected intraperitoneally with gallic acid (100 mg/kg) for 1 week (Mirshekari Jahangiri et al., 2020). Meanwhile, the CCI plus NS group was given the same dose of saline for 1 week.

### Molecular Docking

The protein sequences for P2X3, P2X4, and P2X7 receptors were downloaded from http://www.UniProt.org/, and the P2X.pdb file was obtained by homologous modeling with https://swissmodel.expasy.org/. The gallic acid. sdf file was downloaded from https://pubchem.ncbi.nlm.nih.gov/. The P2X receptor was pretreated with pyMOL software to remove small molecule ligands, dehydrate, and hydrogenate. Then, the gallic acid. sdf file was converted to pdb format. Finally, Autodock Tools soft was employed for molecular docking (Yang et al., 2019).

### Mechanical Withdrawal Threshold and Thermal Withdrawal Latency

Mechanical and thermal hyperalgesia was measured at 2, 4, 6, 8, 10, 12, and 14 days after CCI. The von Frey monofilament test was used to detect the threshold of the MWT (Jia et al., 2017). A BME-410c fully automatic thermal radiation simulator was used to measure the TWL (Yuan et al., 2018). Each rat was measured 6 times with a five-minute interval. All experiments were conducted by experienced researchers in blind manner.

### Quantitative Real-Time PCR

qRT-PCR was performed to determine mRNA levels (Yuan et al., 2018). The sequences of the primers were as follows: P2X7 receptor (forward: 5′-AGC​GTG​AAT​TAC​GGC​ACC​AT-3′, reverse:5′-CAAAGGGAGGGTGTAGTCGG-3′); TNF-α (forward: 5′-CAC​GTC​GTA​GCA​AAC​CAC​CAA-3′, reverse:5′-GTTGGTTGTCTTTGAGATCCAT-3′); GAPDH (forward: 5′-GCG​AGA​TCC​CGC​TAA​CAT​CA-3′, reverse: 5′-CTC​GTG​GTT​CAC​ACC​CAT​CA-3′) and *β*-actin (forward:5′-TGTCACCAACTGGGACGATA-3′, reverse: 5′-GGG​GTG​TTG​AAG​GTC​TCA​AA-3′). The Promega GO Taq Kit (Shanghai Promega Biotech Co., Shanghai, China) was used to produce cDNA as the template for performing RT-PCR analysis on StepOnePlus System (Applied Biosystems, Foster City, CA, United States).

### Western Blotting

The procedure for protein extraction was the same as in a previous study (Yi et al., 2018). Protein samples of 20–30 μg were subject to electrophoresis on SDS polyacrylamide gels. After transferring the proteins to membranes, 5% skimmed milk was used to block at room temperature for 2 h, followed by incubating at 4°C overnight with primary antibodies: anti-P2X7 (Alomone Labs, Jerusalem, Israel), anti-TACE (Novus Biologicals Co., Littleton, United States), anti-TNF-α (Boster Biological Technology, Wuhan, China), anti-NF-κB (Affinity Biosciences, Ohio, United States), anti-STAT3 (Cell Signaling Technology, Beverly, MA, United States), anti-phosphorylated (p)-STAT3 (Cell Signaling Technology, Beverly, MA, United States) or anti-β-actin (ZSGB-BIO, Beijing, China). After washing with TBST for 3 × 10 min, the membranes were incubated at room temperature for 2 h with the second antibodies: goat anti-rabbit IgG (Proteintech, Rosemont, United States), or goat anti-mouse IgG (Proteintech, Rosemont, United States). After 10 min wash with TBST thrice, the membranes were exposed and developed in a gel imaging system. Image-ProPlus 6.0 was used to analyze the results.

### Double-Label Immunofluorescence

The isolated DRG tissues were sliced, washed with PBS buffer for 3 × 5 min, and then fixed in 4% paraformaldehyde solution. The fixed tissues were washed with PBS for 3 × 5 min, blocked with goat serum at 37°C for 1 h, and then incubated with anti-P2X7 (Alomone Labs, Jerusalem, Israel) and anti-GFAP (BioLegend, San Diego, CA, United States) at 4°C overnight. After washes with PBS, the slides were incubated with fluorescent secondary antibodies against goat anti-rabbit tetramethylrhodamine (TRITC) (Affinity Biosciences, Ohio, United States) and goat anti-mouse fluorescein isothiocyanate (FITC) (Affinity Biosciences, Ohio, United States) for 60 min. After washes with PBS, the slides were stained with 4’,6-diamidino-2-phenylindole (DAPI) for 5 min and sealed by anti-fluorescence attenuation agent.

### Enzyme-Linked Immunosorbent Assay

The contents of TACE, TNF-α, and NF-κB in the serum of rats were determined by enzyme-linked immunosorbent assay. The prepared sample and standard were added to the orifice plate and reacted at 37°C for 30 min. Wash the plate five times, add HRP-Conjugated reagent and react for 30 min at 37°C. Wash the plate 5 times, add chromogenic reagent A and B, and react at 37°C for 10 min. Add the stop solution and read the OD value within 15 min.

### Human Embryonic Kidney 293 Cell Culture and Transfection

The culture and transfection of HEK293 cells were conducted as described previously (Yang et al., 2019). FuGENE6 (Shanghai Promega Biotech Co., Shanghai, China) was added to the Opti-MEM medium and the mixture was incubated for 5 min to make a transfection reagent. The pcDNA3.0-EGFP-hP2X7 (P2X7-WT) recombinant plasmid (2.5 μg) labeled with a green fluorescent protein (GFP) was mixed with transfection reagent and incubated for 15 min. Finally, the plasmid-containing medium was added to the culture dish with cells and cultured in an incubator for 24–48 h. The transfection efficiency was observed by fluorescence microscopy and the suitable cells were used for whole cell patch clamp experiments.

### Construction of P2X7 Receptor Mutant Plasmid

The P2X7 receptor point mutation receptor was constructed by pymol. The binding sites of gallic acid and P2X7 were successively mutated into glycine (Gly), and then the mutated receptors were molecularly docked with gallic acid and ATP, respectively. The plasmid of the P2X7 mutant was constructed by selecting sites that could reduce the binding affinity of gallic acid without affecting the ATP. The binding scores of gallic acid and ATP to wild-type and mutant P2X7 receptors were as follow: P2X7-WT: −6.4, −10.1; L97G: −5.6, −10.1; T94G: −6.2, −10.2; P96G: −5.6, −10.2; Q98G: −5.6, −10.2; N292G: −6.7, −10.2; K64G: −6.7, −10.2 (kal/mol). Therefore, L97G mutant was selected for plasmid construction, and the mutant plasmid was provided by Hunan Fenghui Biotechnology Co., Ltd.

### Whole-Cell Patch-Clamp Test

The whole cell patch clamp experiments were performed as described previously (Yang et al., 2019). HEK293 cells transfected with pcDNA3.0-EGFP-hP2X7 wild type (P2X7-WT) and P2X7-pEGFP-C1-MUT mutant (P2X7-Mutant) recombinant plasmid were placed under a microscope, and the perfusion delivery system was modulated at low magnification to get as close to the cell surface as possible. The glass electrode was drawn, filled with about 1/3 of the intracellular fluid, and placed on the electrode gripper. Positive pressure was applied to the electrode which was entering the liquid, sealing and breaking the membrane, thus forming a whole cell mode. At this point, the testing drug was administered through a multi-channel perfusion delivery system and the current was recorded. The concentrations of ATP and gallic acids were 100 μM (Ivetic et al., 2019) and 10 μM (Du et al., 2020), respectively. The current was recorded using Clampex10.3 software.

### Statistical Analysis

SPSS 21.0 software (SPSS, Chicago, IL, United States) and GraphPad Prism7 (GraphPad Software, Inc., La Jolla, United States) were used. Two-way ANOVA and Tukey’s honestly significant difference test were used to analyze the MWT and TWL. Pearson coefficient analysis was used for the results of the double-label immunofluorescence. The other experimental results were analyzed by one-way ANOVA, and the differences among groups were compared by least significant difference (LSD). All results are shown as the mean ± SEM, and *p* < 0.05 indicated that the difference was statistically significant.

## Results

### Molecular Docking of Gallic Acid and P2X Receptors

The molecular docking results showed that the binding affinity of gallic acid to P2X3, P2X4, and P2X7 were −5.6 (kcal/mol) (Table 1), −5.5 (kcal/mol) (Table 2), and −6.4 (kcal/mol) (Table 3), respectively. The binding ability of gallic acid to P2X7 was better by taking the absolute value of affinity >6 kcal/mol as the standard. We chose the P2X7 receptor as the target of gallic acid for subsequent studies.

**TABLE 1:** MOE scores of hP2X3 protein and gallic acid (kcal/mol).

Mode/Rank	Affinity (kcal/mol)	Dist from rmsb* l.b	Best mode rmse u.b
1	−5.6	0	0
2	−5.6	2.575	3.055
3	−5.6	0.461	2.444
4	−5.6	2.940	3.613
5	−5.4	2.785	3.248
6	−4.9	31.182	33.238
7	−4.8	31.423	33.500
8	−4.8	1.850	3.175
9	−4.7	24.734	25.905

**TABLE 2:** MOE scores of hP2X4 protein and gallic acid (kcal/mol).

Mode/Rank	Affinity (kcal/mol)	Dist from rmsb* l.b	Best mode rmse u.b
1	−5.5	0	0
2	−5.1	1.670	2.911
3	−5.1	2.880	5.830
4	−5.0	27.154	28.963
5	−5.0	27.361	29.272
6	−5.0	1.399	1.760
7	−4.8	1.712	3.142
8	−4.7	37.102	38.794
9	−4.6	37.266	38.892

**TABLE 3:** MOE scores of hP2X7 protein and gallic acid (kcal/mol).

Mode/Rank	Affinity (kcal/mol)	Dist from rmsb* l.b	Best mode rmse u.b
1	−6.4	0	0
2	−6.3	2.017	2.860
3	−6.3	2.016	2.719
4	−6.2	16.724	18.833
5	−6.0	2.293	4.321
6	−5.9	15.160	16.896
7	−5.9	15.151	17.025
8	−5.8	18.229	19.853
9	−5.6	2.184	4.406

Explanation: The predicted binding affinity is in kcal/mol (Energy). *rmsd: RMSD was used for cross-sectional comparisons of a set of structurally approximate or related proteins to obtain differences in structural stability under these conditions. Two variants of RMSD metrics are provided, rmsd/lb (RMSD lower bound) and rmsd/ub (RMSD upper bound), representing upper and lower limits of distance or Angle, respectively.

### Effect of Gallic Acid on Pain Behaviors in CCI Rats

Smaller mechanical withdrawal threshold and thermal withdrawal latency indicate greater pain sensitivity in rats. The results in Figures 1B,C showed that 1 week after CCI, the sensitivity of both mechanical and thermal hyperalgesia in CCI group was significantly higher than in the sham group (*p* < 0.001). After treatment with gallic acid, the mechanical and thermal hyperalgesia in CCI rats was significantly reduced (*p* < 0.001). However, no significant difference was observed between the negative control group and the CCI group (*p >* 0.05). These results revealed that gallic acid could significantly alleviate mechanical and thermal hyperalgesia in CCI rats.

### Effect of Gallic Acid on Expression of P2X7 Receptor

**FIGURE 2:**
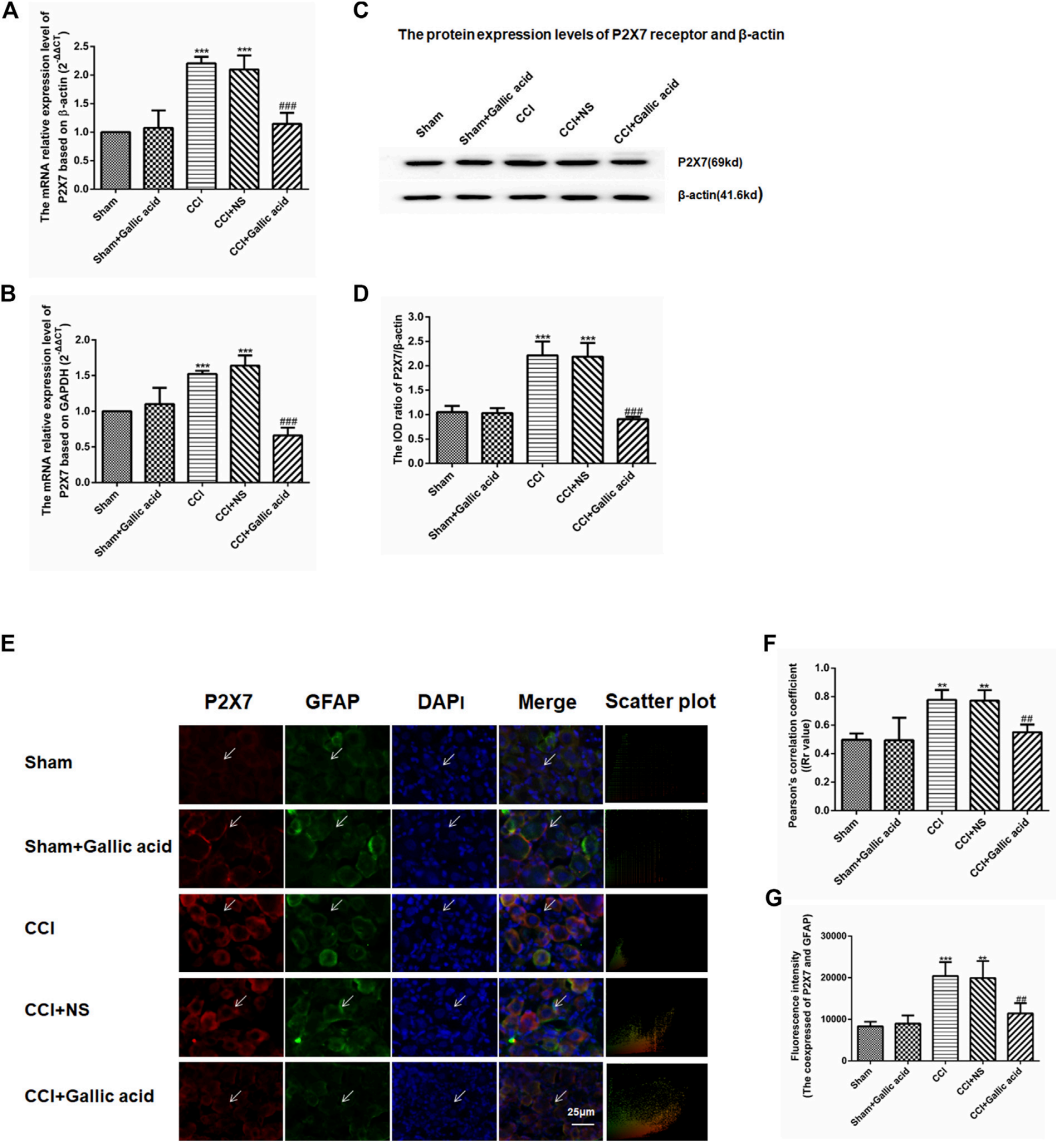
**Effect of gallic acid on the expression of the P2X7 receptor. (A).** The expression of P2X7 mRNA was detected by qRT-PCR using *β*-actin as the housekeeper gene (F (4, 10) = 21.56, *p* < 0.001). **(B).** The expression of P2X7 mRNA was detected by qRT-PCR using GAPDH as the housekeeper gene (F (4, 10) = 49.25, *p* < 0.001). **(C).** The protein expression of P2X7 and *β*-actin in the DRG was detected by Western blotting. **(D).** The relative protein expression of P2X7 receptor (F (4, 10) = 56.34, *p* < 0.001). **(E).** Effect of gallic acid on the coexpression of P2X7 and GFAP in DRG. The coexpression of P2X7 and GFAP in the DRG was detected by a double-label immunofluorescence assay. Arrows indicate active cells. Scale bar: 25 µm. **(F).** Pearson coefficient analysis was used to detect the correlation between P2X7 and GFAP. Rr value represents the degree of correlation between the coexpression of P2X7 and GFAP. Rr is a value between 1 and −1, where one means the variable is completely positively correlated, 0 means irrelevant, and −1 means completely negatively correlated (F (4, 10) = 17.92, *p* = 0.0001). **(G).** The fluorescence intensity of the coexpression of P2X7 and GFAP (F (4, 10) = 17.58, *p* = 0.0002). One-way ANOVA was used to detect the mRNA and protein expression of P2X7 receptors and the fluorescence intensity of coexpression of P2X7 and GFAP. Each group consisted of eight rats. Data are presented as mean ± SEM. ***p < 0.001 versus the Sham group, ^###^ *p* < 0.001 versus the CCI group.

Quantitative real-time PCR used *β*-actin and GAPDH as the housekeeping gene, respectively. Quantitative real-time PCR and western blotting results showed that compared with the sham group, the mRNA and protein levels of the P2X7 receptor in the CCI group were significantly increased (*p* < 0.001). The mRNA and protein levels of P2X7 in the CCI plus gallic acid group were significantly lower than those in the untreated group (*p* < 0.001). However, no significant difference was observed between the CCI plus NS group and the CCI group (*p >* 0.05) (Figures 2A–D). These results suggested that gallic acid could significantly inhibit the expression of the P2X7 receptor at both mRNA and protein levels in CCI rats.

Upregulation of GFAP expression indicates the activation of satellite glial cells (SGCs), thus promotes the release of ATP and inflammatory cytokines, and activates P2X7 receptor. The results of double-label immunofluorescence showed that P2X7 was expressed on SGCs in DRG. Compared with the sham group, the coexpression of P2X7 and GFAP in DRG was significantly increased in the CCI group. The coexpression of P2X7 and GFAP was decreased in CCI plus gallic acid group compared with the untreated group. There was no significant difference between the CCI group and the negative control group. Pearson’s correlation coefficient is a measure of the degree of correlation between two variables. Rr is a value between 1 and −1, where one means the variable is completely positively correlated, 0 means irrelevant, and -1 means completely negatively correlated. Pearson coefficient analysis was used to detect the correlation between P2X7 and GFAP, the colocation scatter plots of P2X7 and GFAP were synthesized by Image-Pro-Plus 6.0 (Figures 2E,F). The fluorescence intensity of the coexpression of P2X7 and GFAP was analyzed by Image-Pro-Plus 6.0 software (Figure 2G). Thus gallic acid could inhibit the coexpression of P2X7 and GFAP in DRG in CCI rats.

### Effect of Gallic Acid on Expression of TACE and TNF-α

**FIGURE 3:**
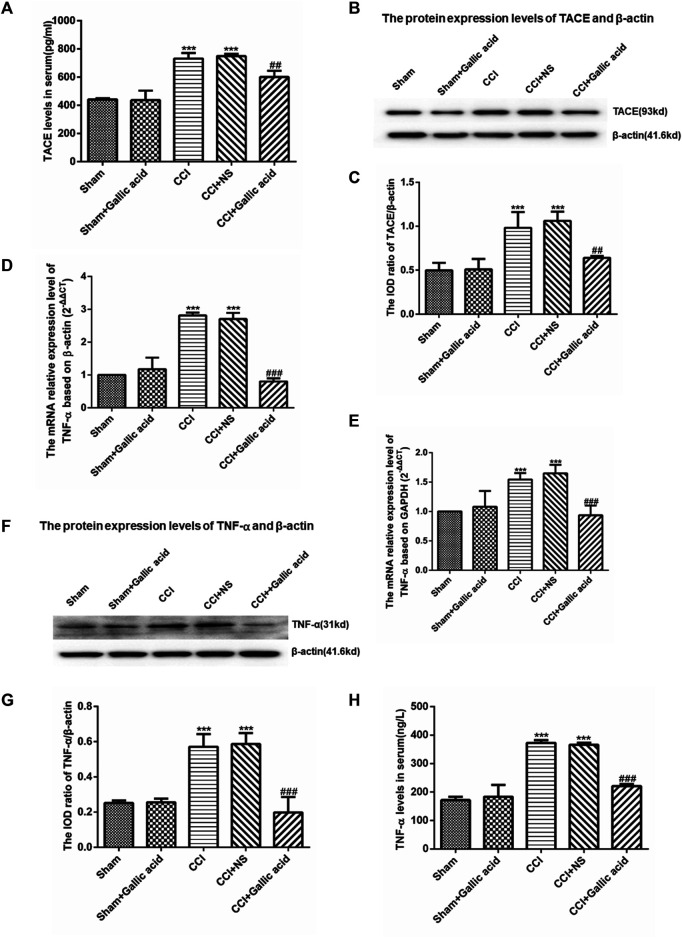
**Effect of gallic acid on the expression of TACE and TNF-α. (A).** The expression of TACE in serum was detected by enzyme-linked immunosorbent assay (F (4, 10) = 89.68, *p* < 0.001). **(B).** Detection of the TACE and *β*-actin protein expression in the DRG by Western blotting. **(C).** The relative protein expression of TACE (F (4, 10) = 24.12, *p* < 0.001). **(D).** The expression of TNF-α mRNA was detected by qRT-PCR using *β*-actin as the housekeeper gene (F (4, 10) = 174.2, *p* < 0.001). **(E).** The expression of TNF-α mRNA was detected by qRT-PCR using GAPDH as the housekeeper gene (F (4, 10) = 22.63, *p* < 0.001). **(F).** In the DRG, TNF-α and *β*-actin protein expression was detected by Western blotting. **(G)**. The relative protein expression of TNF-α (F (4, 10) = 33.47, *p* < 0.001). **(H).** The expression of TNF-α in serum was detected by enzyme-linked immunosorbent assay (F (4, 10) = 212.2, *p* < 0.001). One-way ANOVA was used to detect the expression of TACE and TNF-α. Each group consisted of eight rats. Data are presented as mean ± SEM. ***p < 0.001 versus the Sham group, ^###^ *p* < 0.001 versus the CCI group.

The results of the enzyme-linked immunosorbent assay showed that the contents of TACE and TNF-α in serum were significantly increased in the CCI group compared with the Sham group (*p* < 0.001). The levels of TACE and TNF-α in serum were significantly decreased in the gallic acid treatment group compared with the untreated group (*p* < 0.001). There was no significant difference between the CCI group and the CCI plus NS group (*p >* 0.05) (Figures 3A,H). The TACE protein content of DRG in the CCI group was significantly upregulated compared to the sham group (*p* < 0.001), while it was significantly lower in the CCI plus gallic acid group than that in the untreated CCI group (*p* < 0.001). There was no significant difference between the CCI group and the CCI plus NS group (*p >* 0.05) (Figures 3B,C). In addition, compared with the sham group, TNF-α mRNA and protein levels in DRG of the CCI group were significantly higher (*p* < 0.001); such enhanced expression levels of TNF-α mRNA and protein in DRG were significantly diminished after CCI rats were treated with gallic acid (*p* < 0.001). No significant difference was observed between the CCI group and the CCI plus NS group (*p >* 0.05) (Figures 3D–H). These results indicated that gallic acid could decrease the expression of TACE and TNF-α in CCI rats.

### Effect of Gallic Acid on Expression of NF-κB and STAT3

**FIGURE 4:**
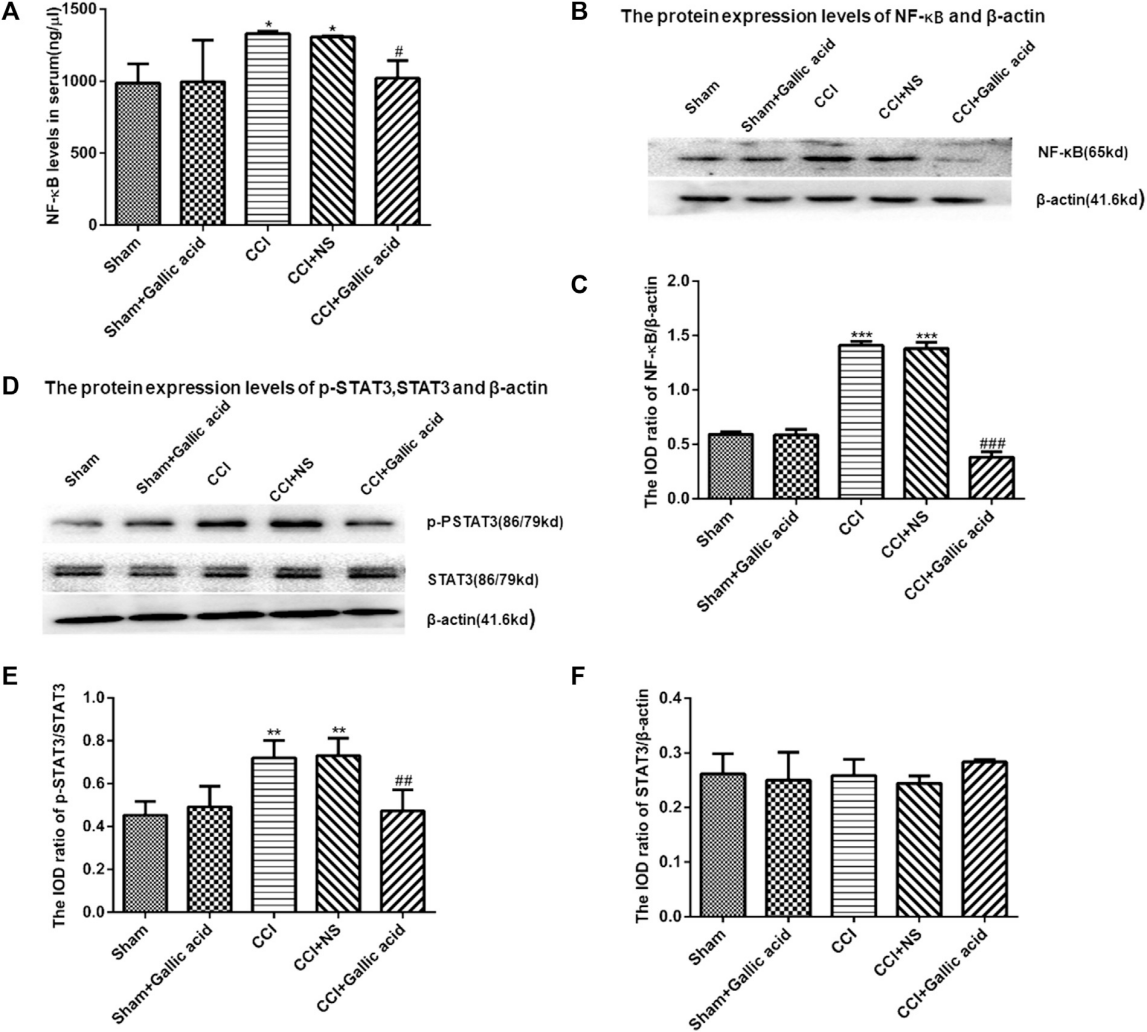
**The effect of gallic acid on the expression of NF-κB and STAT3. (A).** Expression of NF-κB in serum of rats (F (4, 10) = 8.226, *p* = 0.3737). **(B).** The protein expression of NF-κB and *β*-actin in the DRG was detected by Western blotting. **(C).** The relative protein expression of NF-κB (F (4, 10) = 217.4, *p* < 0.001). **(D).** The protein expression of p-STAT3, STAT3, and *β*-actin in the DRG was detected by Western blotting. **(E).** The relative protein expression of p-STAT3 (F (4, 10) = 10.05, *p* = 0.0016). **(F).** The relative protein expression of STAT3 (F (4, 10) = 1.188, *p* = 0.3737). Each group consisted of eight rats. One-way ANOVA was used to detect the expression of NF-κB and STAT3. Data are presented as mean ± SEM. ^**^ *p* < 0.01 and ***p < 0.001versus Sham group, ^##^ *p* < 0.01 and ^###^ *p* < 0.001 versus the CCI group.

The results of the enzyme linked immunosorbent assay showed that the content of NF-κB in the serum of rats in CCI group was significantly higher than that in the Sham group (*p* < 0.001). The content of NF-κB in serum of rats in the CCI plus Gallic acid group was significantly decreased compared with that in the untreated CCI group (*p* < 0.001) (Figure 4A). Compared with the sham group, the protein levels of NF-κB and p-STAT3 in the CCI group were significantly increased (Figures 4B–E). Compared with those in the untreated group, the protein levels of NF-κB and p-STAT3 in the CCI plus gallic acid group were significantly decreased. No significant difference was seen between the CCI group and the negative control group (*p >* 0.05) (Figures 4A–E). There was no significant difference in the expression of STAT3 between the CCI group and the sham group (*p >* 0.05) (Figure 4F). These results suggested that gallic acid could inhibit the expression of NF-κB and p-STAT3 in CCI rats.

### Effect of Gallic Acid on ATP-Activated Current in HEK293 Cells Expressing P2X7

**FIGURE 5:**
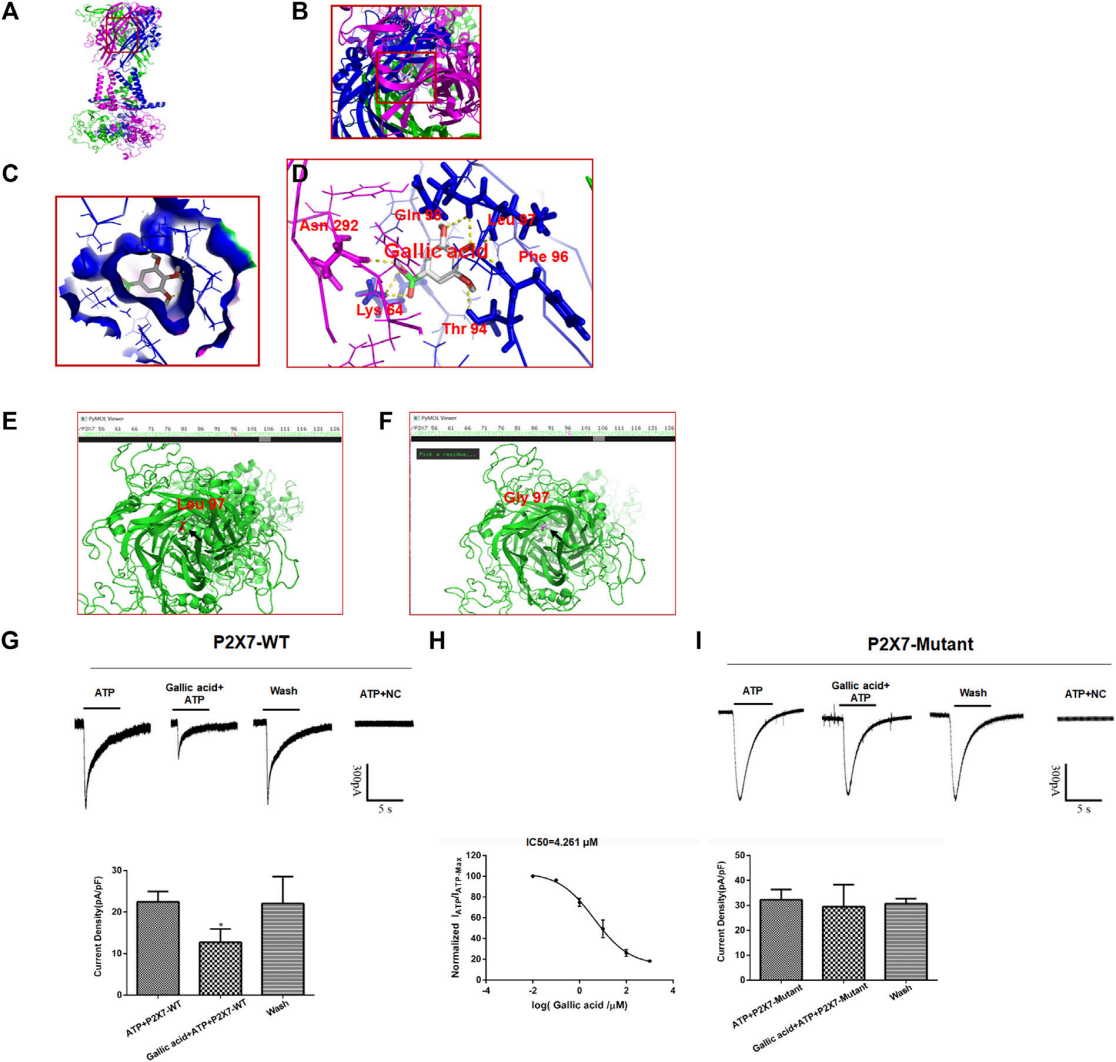
The results of molecular docking and whole-cell patch-clamp. The molecular docking result showed the best binding position between gallic acid and P2X7. The positive view **(A)** and top view **(B)** show the best binding position between gallic acid and P2X7 **(C).** The binding pocket of gallic acid and P2X7 receptor **(D).** The best binding site of gallic acid and P2X7; the yellow dotted line represents the hydrogen bond of the combination of both. The P2X7 receptor mutation diagram shows the mutation site **(E).** The arrow seen in the top view of the wild-type P2X7 receptor indicates a red residue of Leu97 **(F).** The arrow in the top view of the mutant P2X7 receptor indicates that the purple residue is Gly97. Effect of gallic acid on ATP activated current in HEK293 cells transfected with hP2X7 recombinant plasmid **(G).** Effect of gallic acid on ATP-activated current in HEK293 cells transfected with pcDNA3.0-EGFP-hP2X7 (P2X7-WT) was showed (F (2, 6) = 5.634, *p* = 0.0165) **(H).** Inhibition of ATP activation current in HEK293 cells transfected with wild-type P2X7 receptor by different gallic acid concentrations (IC50 = 4.261 μM). **(I).** Effect of gallic acid on ATP-activated current in HEK293 cells transfected with P2X7-pEGFP-C1-MUT (P2X7-Mutant) was showed (F (2, 6) = 0.2648, *p* = 0.7759). The results were analyzed by one-way ANOVA. Data are presented as mean ± SEM, ^*^ *p* < 0.05 versus the ATP alone group.

Molecular docking results showed that gallic acid is bound to a binding pocket composed of P2X7 receptor B and C chains by hydrogen bonding, thus producing interactions with P2X7. As is shown in Figure 5, green represents chain A, purple represents chain B, and blue represents chain C, and A, B, C, and D represent the binding patterns of gallic acid and P2X7 receptors in different fields of vision (Figure 5A). Pymol and AutoDock Tools were used in combination to predict the effects of mutation of the binding sites of gallic acid and P2X7 on the binding affinity of both and the binding affinity of ATP and P2X7. Combined with the results of molecular docking, we finally selected the mutation of Leu97 to Gly97 and constructed the P2X7 mutant for subsequent experiments (Figures 5E,F). ATP-activated currents were recorded by the whole-cell patch-clamp technique. The results showed that gallic acid (10 μM) could significantly inhibit the ATP activation current of HEK293 cells expressing wild-type P2X7 receptor but had a little inhibitory effect on HEK293 cells expressing mutant P2X7 receptor. After washing with extracellular fluid, the recording current returned to the state before gallic acid administration. In addition, the results showed the concentration effect curve of different gallic acid concentrations on the inhibition of ATP activation current in HEK293 cells expressing wild-type P2X7 receptor (IC50 = 4.261 μM) (Figures 5G–I). These results suggest that gallic acid might alleviate neuropathic pain behaviors in CCI rats by inhibiting the P2X7 receptor.

## Discussion

Molecular docking can predict the interaction between drug ligands and receptors (Yang et al., 2019). To determine the direct interaction between gallic acid and P2X7 receptors, we carried out a molecular docking test. The results showed that compared with P2X3 and P2X4 receptors, gallic acid displayed a better affinity to the P2X7 receptor. The docking score (−6.4 kcal/mol) of gallic acid and P2X7 receptor was within a credible range, revealing that there is an interaction between them. Therefore, functional studies for the effects of gallic acid on P2X7 receptors were conducted in an animal model of neuropathic pain.

Neuropathic pain is caused by various central and peripheral injuries. In this study, a classic CCI rat model was used to verify the therapeutic effect of gallic acid on neuropathic pain. Our results show that the MWT and TWL in CCI rats were decreased, and their sensitivity to injury stimulation was increased, which was consistent with previous observations (Zhang et al., 2019). Neuropathic pain is closely related to high levels of proinflammatory cytokines. Gallic acid has analgesic and anti-inflammatory effects (Cao et al., 2019). Indeed, the MWT and TWL in CCI rats were significantly increased after treatment with gallic acid, suggesting that gallic acid relieved the pain behaviors in CCI rats.

The underlying molecular mechanism by which gallic acid alleviates neuropathic behaviors in CCI rats was explored. P2X receptors are involved in neuropathic pain. In particular, P2X3, P2X4, and P2X7 play crucial roles in the treatment of pain (Jacobson et al., 2020). P2X7 is widely expressed in the SGCs of DRG (Neves et al., 2020). Upon activating SGCs after nerve injury, the released ATP and various cytokines from SGCs may act on the P2X7 receptor to affect the pathophysiological processes of neuropathic pain. In this study, mRNA and protein levels of P2X7 were significantly higher in CCI rats, whereas gallic acid treatment could effectively downregulate such enhanced P2X7 expression. In addition, double-label immunofluorescence showed the increased coexpression of P2X7 and GFAP in DRG of CCI rats, and this effect was inhibited after gallic acid treatment. Moreover, Pearson coefficient analysis showed that P2X7 was well correlated with GFAP expression. Therefore, gallic acid reduced the MWT and TWT in CCI rats probably by inhibiting the expression of P2X7 in the activated SGCs of DRG.

TNF-α is an important cytokine and contributes to the pathogenesis of neuropathic pain (Dai et al., 2020). Activated mature TACE can cleave membrane bound TNF-α and convert it into free soluble smaller molecules, which participate in various inflammatory responses and cell signal transduction (Lambertsen et al., 2019). P2X7 promotes the release of mature TACE through exosomes, thus inducing the release of TNF-α (Barbera-Cremades et al., 2017). In this study, the levels of TACE protein, TNF-α mRNA, and protein were upregulated in CCI rats. The contents of TACE and TNF-α in serum were significantly increased in CCI rats. Significantly, these alterations could be downregulated by gallic acid treatment. Thus, gallic acid might inhibit the activation of TACE by interfering with the function of the P2X7 receptor, leading to decreased release of TNF-α to alleviate neuropathic pain behaviors in CCI rats.

NF-κB is an important transcription regulator that exists in almost all mammalian cells. Activated NF-κB can participate in the inflammatory responses (Jimi et al., 2019; Peng et al., 2019; Wu H. et al., 2020). TNF receptor related-factors (TAFRs) are intracellular adaptor proteins that include seven family members (TRAF 1–7). TNF-α can activate NF-κB after binding to TAFR, thereby regulating gene transcription and participating in neuropathic pain (Dou et al., 2018). NF-κB can regulate the transcription of STAT3 and synergistically affect the progression of inflammation (Callejas et al., 2019). Inhibition of the NF-κB/STAT3 signaling pathway can also inhibit acute skin inflammation (Wu J.-Y. et al., 2020). Additionally, the P2X7 receptor can regulate the NF-κB signaling pathway (Cai et al., 2016). In our study, the expression of NF-κB and p-STAT3 was increased in CCI rats, the content of NF-κB in serum of rats in the CCI group was significantly higher than that in the Sham group, indicating the activation of the NF-κB/STAT3 signaling pathway. In contrast, gallic acid treatment could counteract the upregulated expression of NF-κB and p-STAT3 in CCI rats. Hence, reversing the activation of NF-κB and STAT3 signaling pathways subsequent to inhibiting the expression of P2X7 receptor and the release of TNF-α would contribute to the beneficial effects of gallic acid on alleviating mechanical and thermal hyperalgesia in CCI rats.

Electrophysiological experiments can observe the function of receptors. To this, the whole-cell patch-clamp experiments were carried out to analyze the effect of gallic acid on the P2X7 receptor. Moreover, the 3D structures of gallic acid and P2X7 were obtained, and molecular docking results showed that gallic acid binds to a binding pocket composed of six amino acid residues of the P2X7 receptor. The P2X7 receptor mutants were simulated by pymol, and molecular docking was conducted with gallic acid and ATP, respectively. The sites that could reduce the binding affinity of gallic acid but not affect ATP were selected for the construction of the P2X7 receptor site-directed mutant plasmid. The results showed that gallic acid had an inhibitory effect on ATP activation current of HEK293 cells transfected with P2X7-WT plasmid but had no effect on ATP activation current of P2X7-mutant plasmid, indicative of reduced activity of P2X7 receptor. These data further demonstrated that gallic acid could act on the P2X7 receptor to downregulate its function, inhibited the TNF-α/NF-κB/STAT3 signaling pathway in CCI rats.

In conclusion, gallic acid is able to inhibit the activation of SGCs in DRG and alleviate mechanical and thermal hyperalgesia in CCI rats. The underlying molecular mechanisms involve the downregulation of P2X7 receptor expression, reduction of mature TACE release, inhibition of TNF-α expression, and suppression of the NF-κB/STAT3 signaling pathway.

## Data Availability

The original contributions presented in the study are included in the article/Supplementary Material. Further inquiries can be directed to the corresponding author.

## Abbreviations

ATP, adenosine triphosphate; CCI, chronic constriction injury; DRG, dorsal root ganglia; HEK293, human embryonic kidney 293; GFAP, fibrillary acidic protein; SGCs, satellite glial cells; TACE, TNF-α converting enzyme; TNF-α, tumor necrosis factor-α; solTNF-α, soluble TNF-α; tmTNF-α, transmembrane TNF-α; STAT3, signal transducer and activator of transcription 3.
