# Supplementary figures and images for: Gallic Acid Alleviates Neuropathic Pain Behaviors in Rats by Inhibiting P2X7 Receptor-Mediated NF-κB/STAT3 Signaling Pathway
Source: Front Pharmacol. 2021 Aug 25;12:680139. doi: 10.3389/fphar.2021.680139 (PMC8423904; doi:10.3389/fphar.2021.680139)

Sham


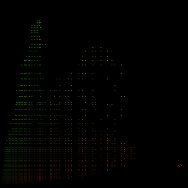


Sham+gallic acid


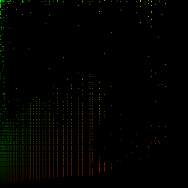


CCI


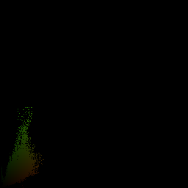


CCI+NS


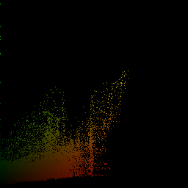


CCI+gallic acid


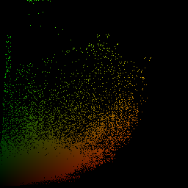

Supplement: Supplementary file 2 [file DataSheet4.docx]

P2X7-WT(Leu97)
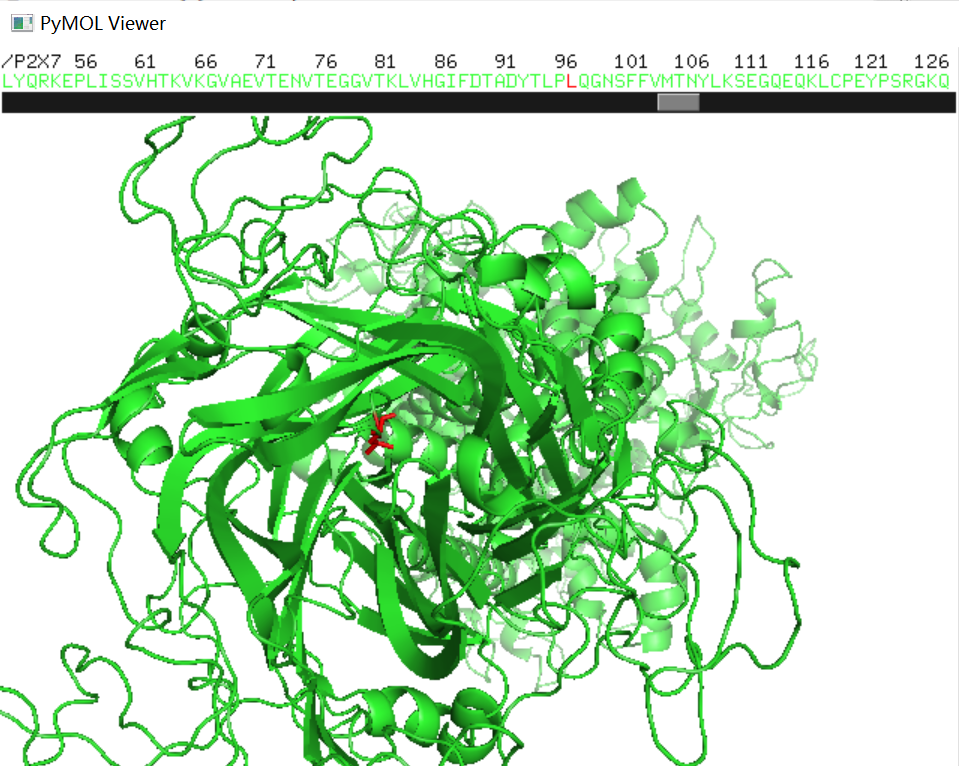


P2X7-Mutant (Gly97)


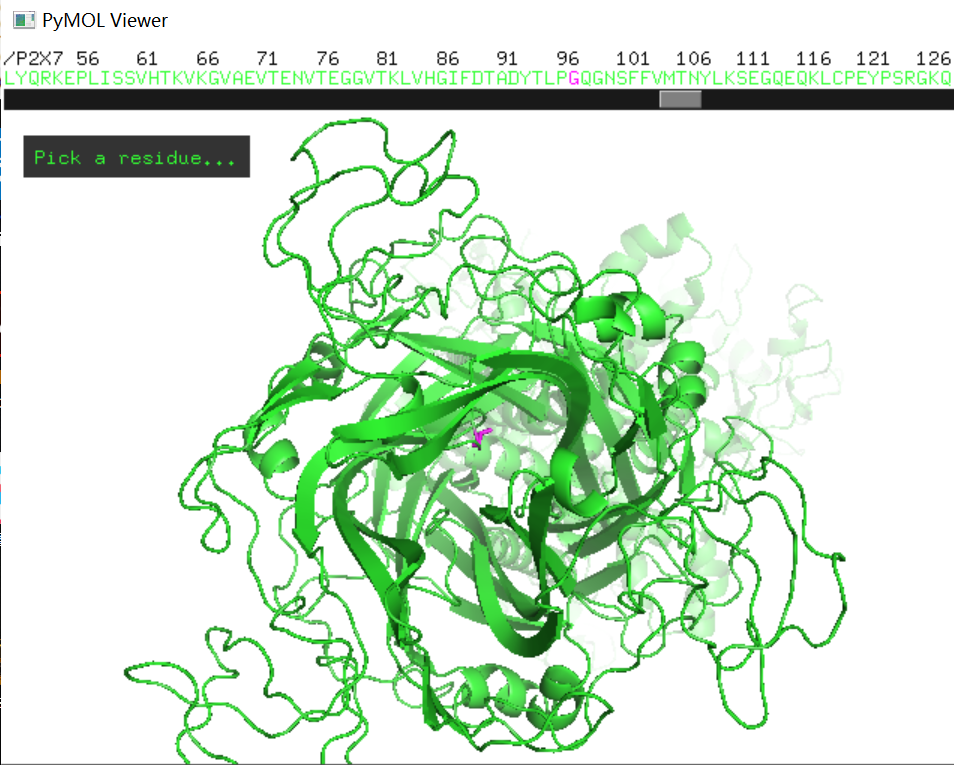

Supplement: Supplementary file 4 [file DataSheet3.docx]

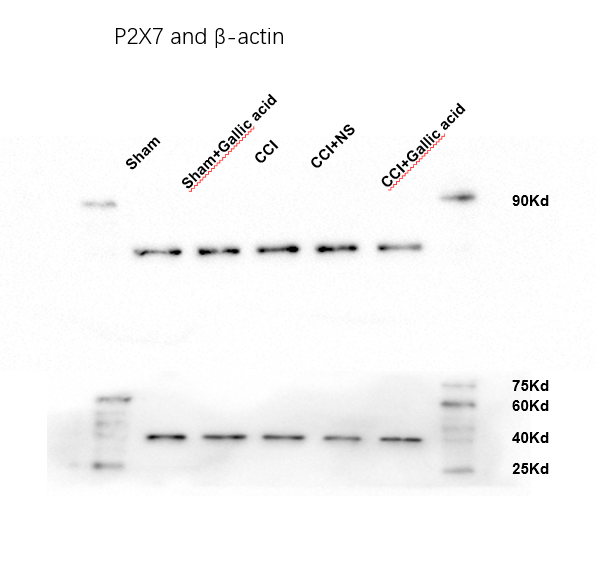


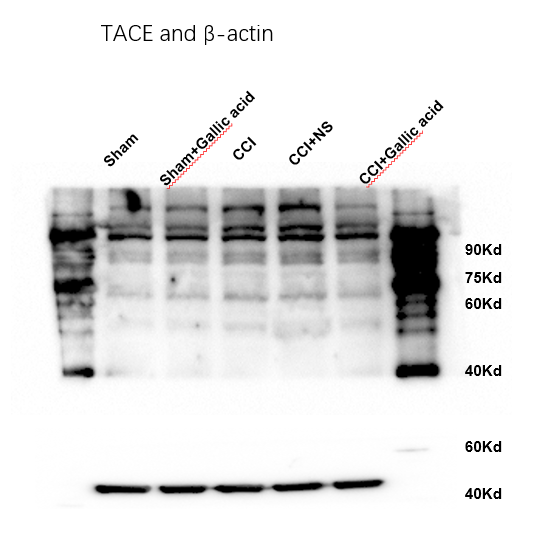


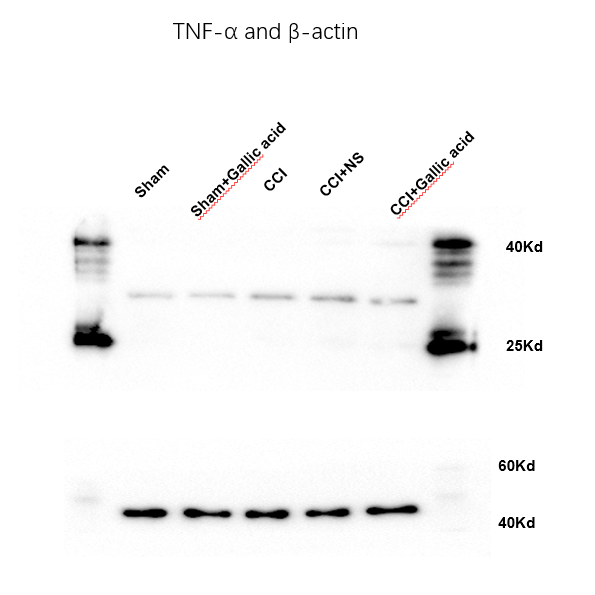


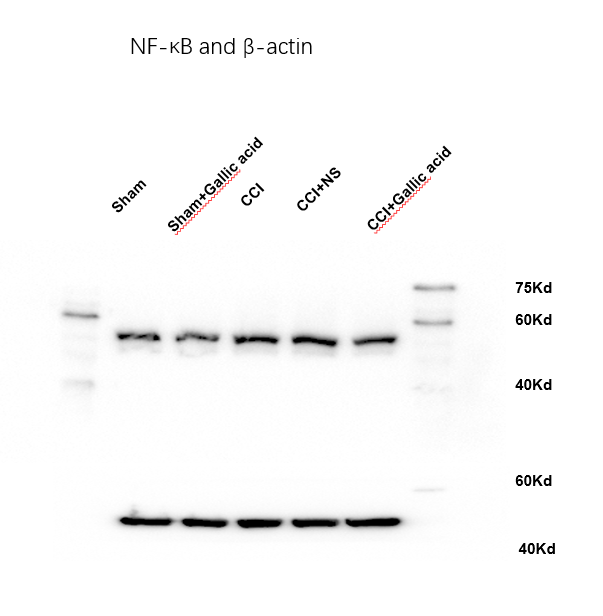


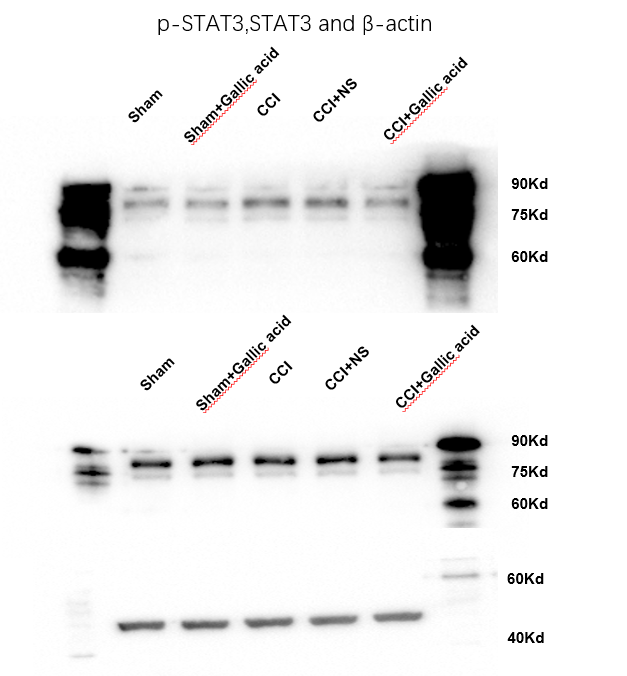

Supplement: Supplementary file 8 [file DataSheet1.docx]
