# Supplementary material for: Gallic Acid Alleviates Neuropathic Pain Behaviors in Rats by Inhibiting P2X7 Receptor-Mediated NF-κB/STAT3 Signaling Pathway
Source: Front Pharmacol. 2021 Aug 25;12:680139. doi: 10.3389/fphar.2021.680139 (PMC8423904; doi:10.3389/fphar.2021.680139)

**MOE score of hP2X7 protein-WT and gallic acid** (kcal/mol)


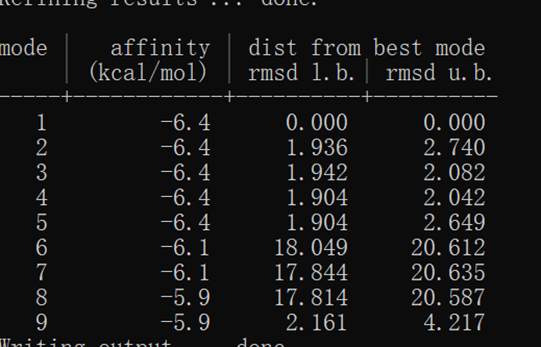


**MOE score of hP2X7 protein-WT and ATP** (kcal/mol)


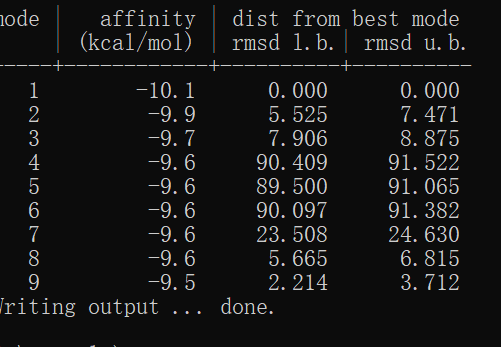


**MOE score of hP2X7 protein-L97G and gallic acid** (kcal/mol)


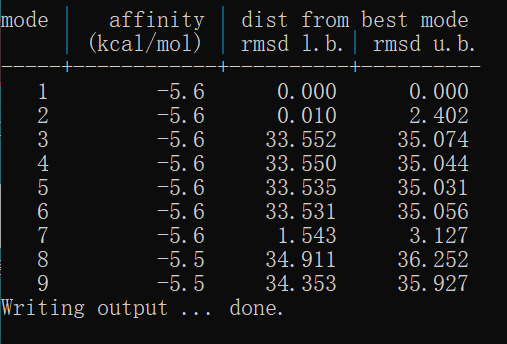


**MOE score of hP2X7 protein-L97G and ATP** (kcal/mol)


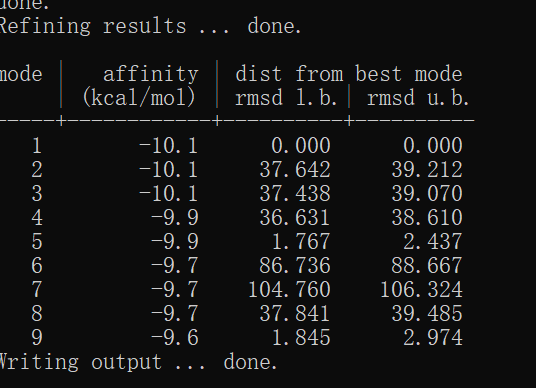


**MOE score of hP2X7 protein-T94G and gallic acid** (kcal/mol)


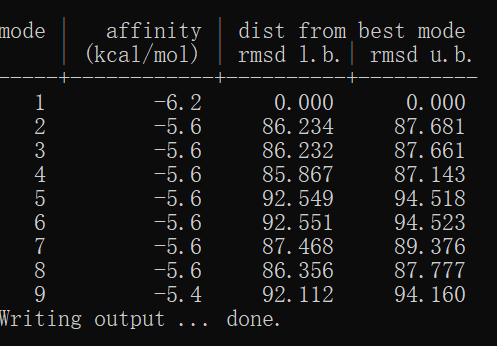


**MOE score of hP2X7 protein-T94G and ATP** (kcal/mol)


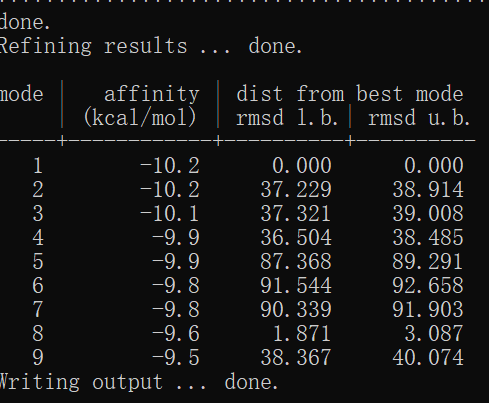


**MOE score of hP2X7 protein-P96G and gallic acid** (kcal/mol)


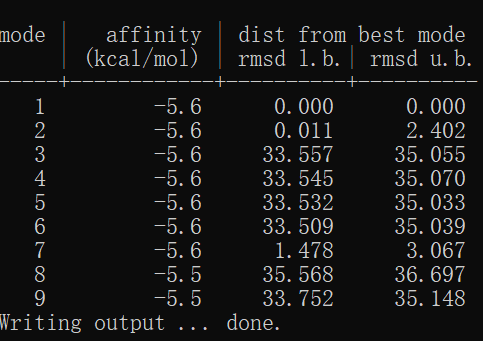


**MOE score of hP2X7 protein-P96G and ATP**(kcal/mol)


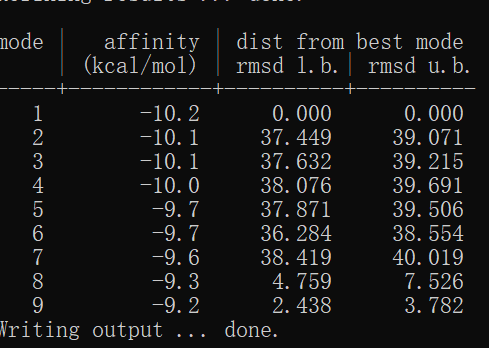


**MOE score of hP2X7 protein-Q98G and gallic acid** (kcal/mol)


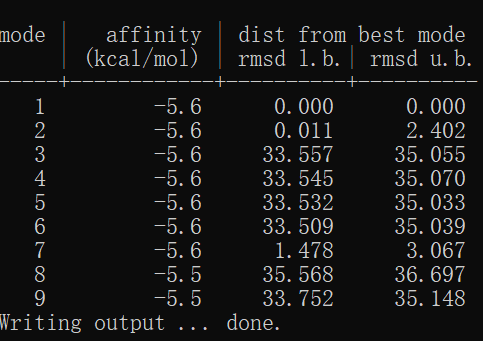


**MOE score of hP2X7 protein-Q98G and ATP** (kcal/mol)


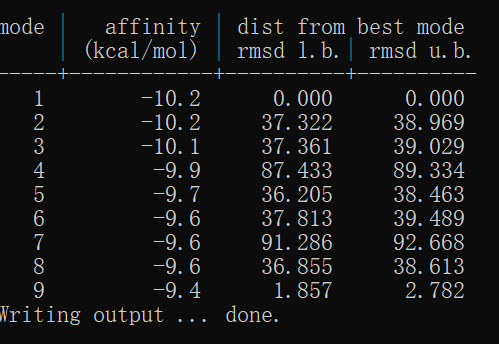


**MOE score of hP2X7 protein-N292G and gallic acid** (kcal/mol)


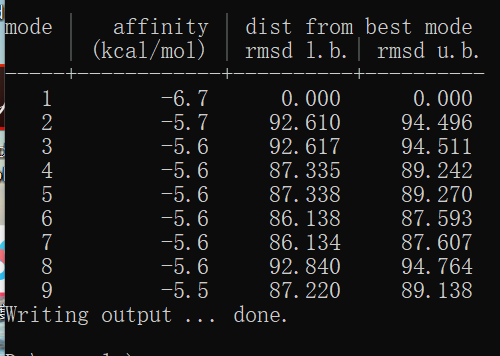


**MOE score of hP2X7 protein-N292G and ATP** (kcal/mol)


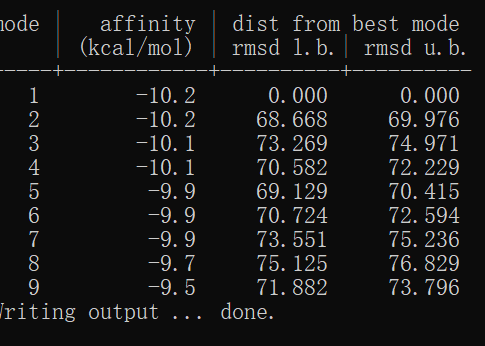


**MOE score of hP2X7 protein-K64G and gallic acid** (kcal/mol)


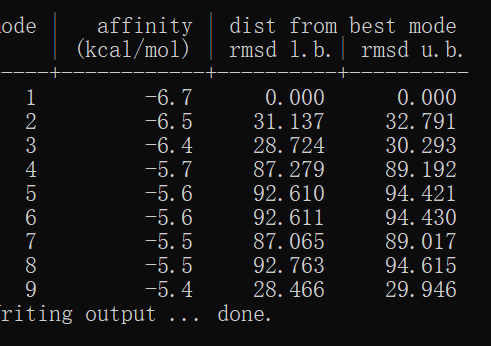


**MOE score of hP2X7 protein-K64G and ATP** (kcal/mol)


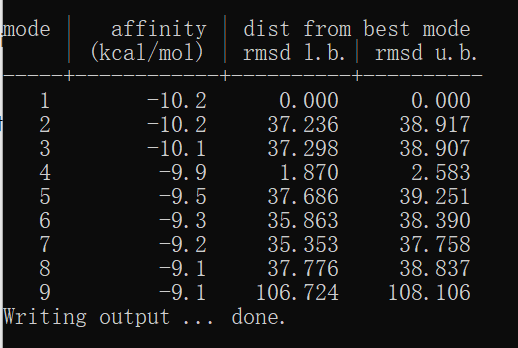

Supplement: Supplementary file 7 [file DataSheet2.docx]
